# Barriers to using new needles encountered by rural Appalachian people who inject drugs: implications for needle exchange

**DOI:** 10.1186/s12954-019-0295-5

**Published:** 2019-04-02

**Authors:** Stephen M. Davis, Alfgeir L. Kristjansson, Danielle Davidov, Keith Zullig, Adam Baus, Melanie Fisher

**Affiliations:** 1 Department of Health Policy, Management, and Leadership, School of Public Health, Robert C. Byrd Health Sciences Center, West Virginia University, PO Box 9190, Morgantown, WV 26506 USA; 2 Department of Emergency Medicine, West Virginia University, PO Box 9149, Morgantown, WV 26506 USA; 3 Department of Social and Behavioral Sciences, West Virginia University, PO Box 9190, Morgantown, WV 26506 USA; 4 Department of Medicine, Section of Infectious Diseases, West Virginia University, PO Box 9163, Morgantown, WV 26506 USA

**Keywords:** Needle exchange programs, Hepatitis C virus, People who inject drugs, Paraphernalia laws, Barriers to using new needles, Policing behaviors

## Abstract

**Background:**

Using a new needle for every injection can reduce the spread of infectious disease among people who inject drugs (PWID). No previous study has examined new needle use barriers among PWIDs residing in the rural Appalachian part of the United States, an area currently in the midst of a heroin epidemic.

**Objective:**

Therefore, our primary aim was to explore self-reported barriers to using a new needle by PWID attending a needle exchange program (NEP).

**Methods:**

We conducted a cross-sectional survey of PWID attending two NEPs in rural West Virginia located in the heart of Central Appalachia. A convenience sample of PWID (*n* = 100) completed the Barriers to Using New Needles Questionnaire.

**Results:**

The median number of barriers reported was 5 (range 0–19). Fear of arrest by police (72% of PWID “agreed” or “strongly agreed”) and difficulty with purchasing needles from a pharmacy (64% “agreed” or “strongly agreed”) were the most frequently cited barriers.

**Conclusions/Importance:**

Congruent with previous findings from urban locations, in rural West Virginia, the ability of PWID to use a new needle obtained from a needle exchange for every injection may be compromised by fear of arrest. In addition, pharmacy sales of new needles to PWID may be blunted by an absence of explicit laws mandating nonprescription sales. Future studies should explore interventions that align the public health goals of NEPs with the occupational safety of law enforcement and health outreach goals of pharmacists.

**Electronic supplementary material:**

The online version of this article (10.1186/s12954-019-0295-5) contains supplementary material, which is available to authorized users.

## Introduction

The United States (US) is currently in the midst of a hepatitis C virus (HCV) epidemic that has been catalyzed by injection drug use in rural areas. Cases of acute HCV infection in the US increased 98% between 2010 and 2015 with consistent annual increases observed during this time period [1]. Approximately 60 to 70% of HCV cases in the US occur in people who inject drugs (PWID) [2, 3]. The prevalence of HCV infection in PWID ranges between 40 and 90% and has been observed to be as high as 98% [4]. A disproportionate number of new HCV cases occur in young, white PWID residing in the rural, Central Appalachian region of the US, which includes the states of Kentucky, Tennessee, Virginia, and West Virginia [1, 2, 5–7]. This region observed a 364% increase in new HCV cases among young persons (aged ≤ 30 years) between 2006 and 2012 [6]. Additionally, in 2015, West Virginia had the second-highest rate of acute HCV infections (3.4 per 100,000) in the US followed by Kentucky (2.7 per 100,000) and Tennessee (2.6 per 100,000) [1]. This exponential increase in HCV cases has been associated with prescription opioid misuse that progresses to injection of heroin [8–11].

Among PWID, the risk of HCV seroconversion increases 94% [pooled risk ratio = 1.94, 95% confidence interval (CI), 1.53, 2.46] when syringes are shared during injection [12]. In an effort to minimize the reuse of needles, needle exchange programs (NEP) have been implemented as a mechanism for exchanging used needles for new needles [10, 13]. Although the ability to access new needles is one important step in fighting the HCV epidemic, there are potential barriers to using new needles obtained from exchange programs that may reduce the efficacy of NEPs.

Previous studies have examined barriers to using a new needle for every injection among PWID in large, diverse urban settings. These studies have consistently found direct associations between fear of law enforcement encounters (e.g., arrest, citation, syringe confiscation), syringe sharing [14–18], and injection equipment sharing [16, 19]. Indirectly, fear of law enforcement encounters has been associated with lack of access to new needles from reduced exchange program participation [17, 20–22], and a reluctance to carry new needles [19, 23, 24], which can increase risky injection behavior [25, 26]. Peer group perceptions [27] and drug withdrawal [14] have also been associated with risky injection behavior. No information currently exists describing the new needle use barriers experienced by PWID in the rural, central Appalachian region of the US that is in the midst of historic heroin and HCV epidemics [28]. In comparison to urban and suburban locations, rural areas have fewer NEPs with less staffing and available budgets that may impact access to new needles [29]. Additionally, conservative states such as West Virginia have histories of favoring a criminal response versus a public health response to drug use, which may increase potential fear of arrest by PWID [30]. Therefore, the aim of this exploratory study was to identify self-reported barriers to using a new needle obtained from an exchange program among PWID attending NEPs in the state of West Virginia, which is located in the heart of Central Appalachia in the US. Improved understanding of these barriers may have implications for the efficacy of needle exchange for the prevention of HCV in rural areas. Based on the previous research [31], we hypothesized that fear of arrest and drug withdrawal would be the most frequently reported barriers to using a new needle.

## Methods

### Design

We conducted a cross-sectional survey of PWID attending the first two NEPs to open in West Virginia, the Cabell-Huntington Health Department Syringe Exchange [32] and the Mylan Puskar Health Right LIGHT (Living in Good Health Together) Program [33]. The Cabell-Huntington Health Department Syringe Exchange is operated by a county health department located in the southern part of West Virginia that has been described as the overdose capital of the US [34], while the LIGHT Program is operated by a free healthcare clinic in another high PWID prevalence area located close to the northern border with Pennsylvania. Both programs exchange used needles for new needles. PWIDs are encouraged to bring all needles back, but the exchange is not dependent on the number of needles returned. At the time of the study, the health department limited the number of needles per PWID due to budgetary constraints. The LIGHT Program did not have a specific limit and distributed new needles on a needs basis. We specifically selected these two NEPs because they were the only programs open at the time of study design and thus had an established client base and study operation flow. Furthermore, the primary author had established relationships with each NEP director, which facilitated survey implementation and logistics.

### Measures

Previous studies examining barriers to new needle use employed variable measurement methods that ranged from qualitative interviews [14], quantitative surveys [19], or mixed methods approaches [15, 35], with different variable operationalizations. To facilitate approximate comparisons to new needle use barriers experienced by urban PWID, we chose to use the Barriers to Using New Needles Survey (Cronbach’s alpha = .84) [31] that was administered to a convenience sample of urban heroin users in Denver, Colorado, in the western US. This questionnaire consists of 20 possible items regarding barriers to using a new needle gleaned from clinical work and qualitative interviews. Each participant indicates the extent to which s/he agrees with each item (barrier) according to a 5-point Likert scale ranging from “strongly disagree” to “strongly agree.”

To test the comprehension and completeness of the survey [36, 37], two focus groups with 8 PWIDs (4 males, 4 females) drawn from attendees at both the Health Department NEP (*n* = 5) and the LIGHT Program (*n* = 3) were conducted. Guiding questions were based on whether (1) items were understandable, (2) response choices were adequate, (3) items were written in a manner that elicited only one response, and (4) items were complete. Item modifications were made as needed to maximize their applicability to the rural setting and avoid poorly defined words or terms that were not universally understood [38]. To further enhance reliability and increase validity, the directors of both NEPs also reviewed these items. All respondents believed the items were complete (i.e., no potential barriers were missed) and written in a manner that only elicited one response. Participants also felt that response choices (i.e., the 5-point Likert scale) were adequate and that most items were understandable with the exception of item 8 that discussed the potential barrier of using a new needle while injecting in a shooting gallery. Several participants were unfamiliar with the term “shooting gallery.” Therefore, we added a clarifying definition and a few terms (i.e., “dope den,” “joy popping”) taken directly from the transcripts of the focus groups that reflect the concept of a shooting gallery in our setting. Otherwise, no other changes were made to the questionnaire (see Additional file 1 for specifics).

After item modifications, basic demographic questions querying age, gender, race/ethnicity, education level, employment status, housing status, HCV status, and injection history (e.g., frequency of injecting, years of injecting) were included in the final questionnaire (see Additional file 1). To minimize potential bias stemming from participant and item nonresponse, the final survey was designed and pilot tested to take no more than 3–5 min to complete [36, 39]. Importantly, to minimize social desirability bias given the sensitive nature of the questions, the survey was anonymous [38].

### Participants and procedure

Based on consultation with the NEP directors, a convenience sample of 100 attendees was planned. This sample size was selected based on the following considerations: (1) NEP director’s assessment of maximum number of accessible participants and (2) the unstable and transient nature of the target population. To promote the number of responses, participants were personally invited by study staff to take the survey while attending the exchange [39]. The only requirements for participation were current exchange attendance and being at least 18 years of age. Those attendees agreeing to participate were given a pen and completed the survey on paper with study staff available to answer any questions regarding survey items. Participants were given a $10 gift card upon completion of the questionnaire. The West Virginia University Institutional Review Board approved the study and waived the requirement for written informed consent given that the only document linking participants to the sensitive and potentially stigmatizing questionnaire would be the consent form.

### Statistical analysis

All questionnaire data were entered into JMP® 13.0 Pro [40]. The median was imputed in cases where respondents gave a range of values instead of a single number (i.e., 5–10 injections = 7.5 injections; 47% of cases) and in the 8% of cases with missing injection history. Frequencies and descriptive statistics were calculated for all measured variables. The extent to which demographic characteristics differed between respondents at each NEP was assessed using chi-square (or Fisher’s exact test when more than 20% of expected cell counts were < 5) for nominal or ordinal variables, and the *t* test (or Wilcoxon test in the case of non-normally distributed outcomes) for continuous variables. Normality of outcomes was assessed via the Shapiro-Wilk *W* test. Continuous variables that significantly departed from normality were recoded into an ordinal variable based on distribution quartiles and a goal of creating category cell sizes that met the chi-square test requirement of no more than 20% of cells with expected counts < 5.

#### Barriers to using new needles

The 5-point Likert response indicating agreement with each barrier was collapsed into a dichotomous “agree” (responses of “agree,” “strongly agree”) and “disagree” (responses of “strongly disagree,” “disagree,” “neutral”) variable for analysis to allow comparisons with a previous cohort of Denver heroin injectors that completed the Barriers to Using New Needles Questionnaire [31]. Contingency table analysis was used to explore bivariate associations between nominal and ordinal demographic and injection history factors and (1) the number of barriers reported (< 4, 4–8, > 8) and (2) agreement (i.e., responses of “agree,” “strongly agree”) with the individual barriers to new needle use that were endorsed by more than 50% of respondents. Analysis of variance (ANOVA) was used to examine associations between age and reported barriers. An alpha of .05 was selected as the threshold for statistical significance.

## Results

### Participants

Overall, 100 PWIDs (Health Department, *n* = 74; LIGHT Program, *n* = 26) were approached and agreed to take the modified questionnaire. Respondents were generally young (i.e., < 40 years; range = 18–63 years) and non-Hispanic White with slightly more males than females completing the questionnaire. Most respondents (78%) reported no education beyond high school, and two thirds (66%) were either unemployed or unable to work. A substantial portion of respondents (40%) reported that they were currently homeless and one third were HCV positive. Respondents reported injecting between 1 and 18 times a day, 2 to 150 times a week, for 1 month to 35 years. There were no statistically significant differences between NEP sites in any of the demographic variables measured (see Table 1).

**Table 1:** Participant characteristics

Demographics	All (*n* = 100)		Health department (*n* = 74)	LIGHT Program (*n* = 26)		P*
Age (avg.)	37.05		37.97	34.42		.1307
	%	*n*	%	*n*	%	
Gender						
Female	43	33	44.60	10	38.46	
Male	57	41	55.40	16	61.54	.5869
Education						
< High school	21	19	25.68	2	7.69	
High school graduate	57	39	52.70	18	69.23	
> High school	22	16	21.62	6	23.08	.1420
Race						
White	96	71	95.95	25	96.15	
Black/African American	4	3	4.05	1	3.85	1.000
Hispanic						
Yes	1	1	1.35	0	0	
No	98	72	97.30	26	100	
Missing	1	1	1.35	0	0	1.000
Employment status						
Employed	22	15	20.27	7	26.92	
Unemployed	42	30	40.54	12	46.15	
Unable to work	24	20	27.03	4	15.39	
Other (e.g., student)	12	9	12.16	3	11.54	.6539
Homeless						
Yes	40	33	44.60	7	26.92	
No	60	41	55.40	19	73.08	.1136
Hepatitis C positive						
Yes	32	25	33.78	7	26.92	
No	58	41	55.41	17	65.39	
Do not know	10	8	10.81	2	7.69	.6702
Injection history						
Daily injections (median)	5		5	5		.6776
Weekly injections (median)	30		30	31.25		.5678
Duration injecting (years)	4		4	5.25		.0612
Barriers to new needle use						
No. of barriers (median)	5		5.5	5		.7227
Most frequently reported barrier	Fear of arrest		Fear of arrest	Fear of arrest		

*Test for the difference between the Health Department and the LIGHT Program

### Drug of choice

Drug of choice information was available for 94 participants. Heroin was the most frequently cited drug of choice by respondents (*n* = 67, 71.27%). Other drugs of choice included methamphetamine (*n* = 16, 17.02%), buprenorphine (*n* = 11, 11.70%), cocaine (*n* = 2, 2.13%), fentanyl (*n* = 1, 1.06%), and amphetamines (*n* = 1, 1.06%).

### Barriers to using new needles

#### Number of barriers reported

The median number of barriers to using new needles among all participants was 5 (range 0–19). PWID who reported > 8 barriers were significantly older than those reporting < 4 barriers (41.87 years vs. 35.68 years, respectively; *P* = .02). PWID who had been injecting for at least 8 years were significantly more likely to report > 8 barriers to new needle use compared to those injecting for < 3 years (41.67% versus 12.50%, respectively; *P* = .048). There were no other significant differences (all *P* > .05) in the number of barriers reported between sites, demographic categories, or by frequency of injection.

#### Barriers reported by more than half of respondents

Among all 20 barriers, three items were endorsed by at least 50% of respondents (see Table 2). Two of these barriers related to obtaining new needles at pharmacies (items 11 and 13), and one reflected fear of arrest (item 20). Fear of arrest was the most frequently cited barrier overall with 45% of respondents strongly agreeing and an additional 27% agreeing that it was a barrier to using new needles. Agreement with these three items did not differ significantly (all *P* > .05) by age, gender, race, ethnicity, education, employment status, housing status, HCV status, or injection history. Therefore, we did not construct additional multivariate models potentially controlling for confounders.

**Table 2:** Reported barriers to using new needles by people who inject drugs (*N* = 100)

	Agree	Disagree/neutral
	%	%
1. It takes too long to get a new needle every time I inject.	15	85
2. It is inconvenient to get a new needle every time I inject.	24.74	75.26
3. I often do not want to take the time to get a new needle because my cravings or urges to use drugs are too strong.	18.37	81.63
4. I often do not take the time to get a new needle if I am drug sick or in withdrawal.	24.45	75.55
5. I do not take the time to get a new needle before injecting because I can only think about getting high.	23.23	76.77
6. I do not take the time to get a new needle before injecting if I am already high or drunk.	19.39	80.61
7. The places where I inject usually do not have access to new needles.	29.29	70.71
8. If I am in a shooting gallery (a place where people inject drugs, “dope den,” “joy popping,” etc.), I often do not use a new needle.	21.74	78.26
9. I often do not carry new needles with me when I am out.	30.93	69.07
10. There is not a needle exchange close by for me to get needles.	16.67	83.33
11. Pharmacies sometimes give me hassle when I try to buy needles.	63.33	36.37
12. After I inject, I do not prepare in advance by getting new needles ready for my next injection.	39.80	60.20
13. It’s too expensive to buy new needles from the pharmacy for every time I inject.	55.91	44.09
14. Feeling sad or depressed would get in the way of my using a new needle every time I inject.	20.21	79.79
15. It is embarrassing to buy needles at the pharmacy.	47.92	52.08
16. I worry that someone (friends, family, etc.) may see me buying needles at the pharmacy.	49.47	50.53
17. My peers/friends would look at me funny if I used a new needle every time I inject.	17.53	82.47
18. Having to worry about using a new needle interrupts the ritual of using.	16.84	83.16
19. I am unlikely to use a new needle if a friend lets me borrow his or her used needle.	19.59	80.41
20. I could get in trouble from the police if I carry needles around with me.	72.00	28.00

## Discussion

We conducted the first study to empirically assess barriers to using new needles by PWID living in the Appalachian region of the US that is currently in the midst of an opioid and HCV epidemic. As hypothesized, similar to previous studies, fear of arrest by law enforcement officials was the most frequently reported barrier to using new needles in our population. However, we did not find being in withdrawal to be a significant barrier to new needle use as we expected. Rather, difficulty obtaining new needles from pharmacies was the second most frequently cited barrier. These findings may have implications for the efficacy of NEPs for the prevention of HCV and other infectious diseases among PWID.

The overall number of barriers reported was slightly larger in the Denver sample compared to our study (average of 7.19 (SD, 3.62) barriers reported versus 6.02 (SD, 4.61), respectively). However, similar to our findings, only a small proportion of barriers was endorsed by at least 50% of respondents. More specifically, only 5 of 20 barriers were endorsed by more than 50% of respondents in Denver, and only 3 of 20 barriers were endorsed by more than 50% in our rural setting, although an additional 2 barriers related to difficulties purchasing new syringes at a pharmacy (items 15 and 16) were endorsed by almost half of our sample.

Notably, and congruent with our findings, fear of arrest for carrying new needles was the most frequently cited barrier in Denver (98% of injectors, 45 of 48). This finding is also congruent with several other studies conducted in the following large, urban areas: Baltimore, MD [19, 41], Oakland, CA, Richmond, CA [16], Los Angeles, CA [14, 15], and San Francisco, CA [42], in the US and Moscow, Volgograd, and Barnaul in Russia [23]. To our knowledge, this work is the first to quantitatively assess this phenomenon in rural Appalachia and is congruent with a recent qualitative case study of rural Appalachian NEPs by Davis et al. [43]. This case study observed a legal conundrum created by paraphernalia laws that manifested in PWID being cited for new needle possessions. West Virginia state law does not regulate the retail sale of syringes [44]. However, some West Virginia cities are passing ordinances that criminalize needle possession in the absence of a prescription [45]. This development may limit the ability of PWID to have a new needle available for every injection, which may increase the risk of HCV seroconversion and undermine the infectious disease prevention goals of NEPs.

Even in locations that do not prohibit syringe possession, the success of NEPs is directly contingent on close cooperation between law enforcement and public health officials. In particular, Beletsky [46] noted a discrepancy between the “law on the books” (i.e., legality of syringe possession and exchange) and the “law on the streets” (i.e., discretionary behavior of law enforcement in the form of arrests and citations for needle possession) [46]. Therefore, a better understanding of law enforcement attitudes underlying variable enforcement behaviors is indicated. Elucidation of these attitudes can form the basis of tailored interventions to promote law enforcement acceptance of NEPs at a local or regional level to improve success and acceptability [47].

Interestingly, the difficulties related to purchasing syringes at pharmacies observed in our study were not reported in the urban, Denver sample, although the legality of syringe sales was similar to our own setting (i.e., no explicit law prohibiting nonprescription sales to PWID) [48, 49]. Therefore, these sales are left to the discretion of pharmacists who often have to weigh the public health goal of preventing infectious disease with concerns over endorsement of drug use, which may contribute to practice variability (Garofoli M, e-mail communication, 7 Nov 2017). Such variability has been documented in the US with syringe purchase refusal rates between 23 and 79% observed in states where nonprescription sale of syringes by pharmacies is legal [50–52]. Concerns over endorsement of drug use and stigmatization of drug users were among the factors associated with these refusals [50]. As a result of these findings, there has been a call to pass legislation that explicitly mandates pharmacy sales of nonprescription syringes as opposed to current discretionary practices [50, 53]. Education of pharmacists to promote awareness of existing state laws that allow nonprescription sales of new needles and the efficacy of new needle use in preventing infectious diseases has been advanced [50, 53]. Pharmacy policies that explicitly promote nonprescription syringe sales have been encouraged [54]. Fuller et al. [55] implemented a multilevel community-based intervention that was successful in reducing negative opinions of pharmacy syringe sales to PWID among both community members and pharmacists. This study also observed increased purchase of syringes by PWID and a significant decrease in syringe reuse. Given the recent closing of a West Virginia NEP over public concerns regarding discarded needles [56], and the fact that the 4 of the top 5 barriers were related to pharmacy sales (i.e., Q11, Q13, Q15, Q16), pharmacy syringe distribution to PWID may be a viable alternative source of new needles and is a topic worthy of additional inquiry and research.

Finally, we did not find being in withdrawal to be a barrier to using new needles in our rural sample as was observed in Denver. Whereas only one quarter of our respondents cited being in withdrawal as a barrier to using new needles in our setting, 65% either agreed or strongly agreed that this was a barrier in Denver, Colorado. Keyes et al. [57] has hypothesized a greater availability of opioids in rural areas due to tight social networks and kinship that serve as a source of prescription opioids. This hypothesis may partially explain the lack of association with withdrawal in our setting. However, it is unclear if this tight social network also increases the availability of nonprescription, illicit opioids (i.e., heroin) that were the most reported drugs of choice in the current study. Therefore, potential explanations for this discrepancy are unclear and warrant further investigation. It is also unclear whether these tight social networks increase access to new needles in the form of secondary exchange from NEP attendees. Des Jarlais et al. [29] reported that an estimated 30% of syringes obtained from rural NEPs are distributed via secondary exchange/peer delivery services. However, these data were reported by NEP directors and not the NEP attendees themselves.

Our findings are subject to some limitations. The results may be subject to reporting bias, specifically in the form of social desirability bias. However, we employed an anonymous survey design to minimize this source of bias. Additionally, given the hard-to-reach PWID population, we were not able to employ a random sampling scheme, which may have biased our results. Such bias could affect the generalizability of our findings both to other injectors attending the two NEP sites in our study and also to PWID attending other NEPs located within West Virginia (9 additional NEPs have opened in West Virginia [58], since the first two that were selected for this study). Our results may also underestimate barriers to new needle use due to the fact that we only recruited PWID attending needle exchanges where some needle access is provided. Although we found no significant differences between participants at both NEPs on selected characteristics, the variable program characteristics of the NEPs included in our study (i.e., one operated by a health department and one operated by a free healthcare clinic) may have contributed to differential participation patterns by PWID that may limit generalizability of our findings. Furthermore, these results may not be applicable to PWID in other locales outside of the rural, Appalachian environment or even to other PWID in West Virginia. Finally, our primary findings are descriptive and could be subject to confounding effects.

These limitations notwithstanding, we were able to provide heretofore-unknown information on new needle use barriers experienced by PWID at the epicenter of the current national HCV epidemic. Future studies should measure the impact of reported barriers on risky injection behavior and seek to confirm these results in other locales. Future studies should also specifically examine the impact of paraphernalia laws and policing behaviors on the efficacy of the needle exchange model in rural Appalachian settings.

## Conclusions

Fear of arrest for possessing new needles and difficulties with pharmacy purchase of new needles were the most commonly cited barriers to using new needles among our population of injection drug users attending two NEPs in rural West Virginia. Future studies should confirm these results in other rural settings and explore the design and implementation of individual and structural interventions with law enforcement officials and pharmacists that promote access to sterile syringes and support the overall efficacy of the needle exchange model.

## Additional file

Additional file 1: Barriers to Using New Needles Questionnaire. (DOCX 24 kb)

## Abbreviations

CA: California
HCV: Hepatitis C virus
MD: Maryland
NEP: Needle Exchange Program
PWID: People who inject drugs
US: United States

## References

[1] CDC (2017). Viral hepatitis surveillance - United States, 2015.

[2] Ward JW (2013). The hidden epidemic of hepatitis C virus infection in the United States: occult transmission and burden of disease. Top Antivir Med.

[3] Schoener EP, Hopper JA, Pierre JD. Injection drug use in North America. Infect Dis Clin North Am. 2002;16(3):535-51, vii. Review. PubMed PMID: 12371114. 10.1016/s0891-5520(02)00010-7 12371114

[4] Hagan H, Pouget ER, Des Jarlais DC (2011). A systematic review and meta-analysis of interventions to prevent hepatitis C virus infection in people who inject drugs. J Infect Dis.

[5] Suryaprasad AG (2014). Emerging epidemic of hepatitis C virus infections among young nonurban persons who inject drugs in the United States, 2006–2012. Clin Infect Dis.

[6] Zibbell JE (2015). Increases in hepatitis C virus infection related to injection drug use among persons aged </=30 years - Kentucky, Tennessee, Virginia, and West Virginia, 2006–2012. MMWR Morb Mortal Wkly Rep.

[7] Havens JR (2013). Individual and network factors associated with prevalent hepatitis C infection among rural Appalachian injection drug users. Am J Public Health.

[8] Moody L, Satterwhite E, Bickel WK (2017). Substance use in rural central Appalachia: current status and treatment considerations. Rural Ment Health.

[9] Fraser H, Zibbell J, Hoerger T, Hariri S, Vellozzi C, Martin NK, Kral AH, Hickman M, Ward JW, Vickerman P. Scaling-up HCV prevention and treatment interventions in rural United States-model projections for tackling an increasing epidemic. Addiction. 2018;113(1):173-82. 10.1111/add.13948. Epub 2017 Sep 20. PubMed PMID: 28734093; PubMed Central PMCID: PMC6211174. 10.1111/add.13948 PMC6211174 28734093

[10] Des Jarlais DC (2017). Harm reduction in the USA: the research perspective and an archive to David purchase. Harm Reduct J.

[11] Zibbell JE (2018). Increases in acute hepatitis C virus infection related to a growing opioid epidemic and associated injection drug use, United States, 2004 to 2014. Am J Public Health.

[12] Pouget ER, Hagan H, Des Jarlais DC (2012). Meta-analysis of hepatitis C seroconversion in relation to shared syringes and drug preparation equipment. Addiction.

[13] Fernandes RM (2017). Effectiveness of needle and syringe programmes in people who inject drugs - an overview of systematic reviews. BMC Public Health.

[14] Wagner KD (2010). The perceived consequences of safer injection: an exploration of qualitative findings and gender differences. Psychol Health Med.

[15] Wagner KD, Simon-Freeman R, Bluthenthal RN (2013). The association between law enforcement encounters and syringe sharing among IDUs on skid row: a mixed methods analysis. AIDS Behav.

[16] Bluthenthal RN (1999). Drug paraphernalia laws and injection-related infectious disease risk among drug injectors. J Drug Issues.

[17] Wood EF (2017). Differential experiences of Mexican policing by people who inject drugs residing in Tijuana and San Diego. Int J Drug Policy.

[18] Pollini RA (2008). Syringe possession arrests are associated with receptive syringe sharing in two Mexico-US border cities. Addiction.

[19] Flath N (2017). Enduring consequences from the war on drugs: how policing practices impact HIV risk among people who inject drugs in Baltimore City. Subst Use Misuse.

[20] Beletsky L (2014). Syringe access, syringe sharing, and police encounters among people who inject drugs in New York City: a community-level perspective. Int J Drug Policy.

[21] Bluthenthal RN (1997). Impact of law enforcement on syringe exchange programs: a look at Oakland and San Francisco. Med Anthropol.

[22] Davis CS (2005). Effects of an intensive street-level police intervention on syringe exchange program use in Philadelphia, PA. Am J Public Health.

[23] Sarang A, Rhodes T, Platt L (2008). Access to syringes in three Russian cities: implications for syringe distribution and coverage. Int J Drug Policy.

[24] Grund JP (1995). In eastern Connecticut, IDUs purchase syringes from pharmacies but don’t carry syringes. J Acquir Immune Defic Syndr Hum Retrovirol.

[25] Cox J (2009). Access to sterile injecting equipment is more important than awareness of HCV status for injection risk behaviors among drug users. Subst Use Misuse.

[26] Bluthenthal RN (2004). Sterile syringe access conditions and variations in HIV risk among drug injectors in three cities. Addiction.

[27] Bailey SL (2007). Perceived risk, peer influences, and injection partner type predict receptive syringe sharing among young adult injection drug users in five U.S. cities. Drug Alcohol Depend.

[28] Davis SM (2017). Needle exchange programs for the prevention of hepatitis C virus infection in people who inject drugs: a systematic review with meta-analysis. Harm Reduct J.

[29] Des Jarlais DC (2015). Syringe service programs for persons who inject drugs in urban, suburban, and rural areas - United States, 2013. MMWR Morb Mortal Wkly Rep.

[30] Cloud DH (2018). Syringe decriminalization advocacy in red states: lessons from the North Carolina harm reduction coalition. Curr HIV/AIDS Rep.

[31] Phillips KT (2015). Barriers to practicing risk reduction strategies among people who inject drugs. Addict Res Theory.

[32] North American Syringe Exchange Network (2019). Cabell-Huntington Harm Reduction Program.

[33] Milan Puskar Health RIGHT (2017). LIGHT Program: Living In Good Health Together (LIGHT).

[34] Quinones S. Dreamland: the true tale of America’s opiate epidemic 2016. Broadway: Bloomsbury Press. https://www.amazon.com/Dreamland-True-Americas-Opiate-Epidemic/dp/1620402521/ref=tmm_pap_swatch_0?_encoding=UTF8&qid=1553714173&sr=8-1.

[35] Wagner KD (2011). The influence of the perceived consequences of refusing to share injection equipment among injection drug users: balancing competing risks. Addict Behav.

[36] Fowler F (2014). Evaluating survey questions and instruments. Survey research methods.

[37] Australian Bureau of Statistics. Pre-testing in survey development: an Australian Bureau of Statistics perspective. 2001. Available from: https://www.abs.gov.au/websitedbs/D3310114.nsf/home/Basic+Survey+Design+-+Survey+Testing. Accessed 5 Nov 2017.

[38] Fowler F (2014). Designing questions to be good measures. Survey research methods.

[39] Fowler F (2014). Implementing a sample design.

[40] SAS Institute, I (2017). JMP Pro 13.0.

[41] Latkin CA (1995). Self-reported reasons for needle sharing and not carrying bleach among injection drug users in Baltimore, Maryland. J Drug Issues.

[42] Bluthenthal R (1999). Collateral damage in the war on drugs: HIV risk behaviors among injection drug users. Int J Drug Policy.

[43] Davis SM (2018). Qualitative case study of needle exchange programs in the Central Appalachian region of the United States. PLoS One.

[44] Project., L.A (2018). Syringe distribution laws. Policy Surveillance Program.

[45] Delancey S (2017). Hurricane Town Council votes on anti-needle ordinance.

[46] Beletsky L, Macalino GE, Burris S (2005). Attitudes of police officers towards syringe access, occupational needle-sticks, and drug use: a qualitative study of one city police department in the United States. Int J Drug Policy.

[47] Beletsky L (2011). Police training to align law enforcement and HIV prevention: preliminary evidence from the field. Am J Public Health.

[48] CDC (2018). Laws related to the retail sale of syringes/needles.

[49] Campbell CA (2017). State HCV incidence and policies related to HCV preventive and treatment services for persons who inject drugs - United States, 2015–2016. MMWR Morb Mortal Wkly Rep.

[50] Pollini RA, Rudolph AE, Case P (2015). Nonprescription syringe sales: a missed opportunity for HIV prevention in California. J Am Pharm Assoc (2003).

[51] Compton WM (2004). A multistate trial of pharmacy syringe purchase. J Urban Health: Bull N Y Acad Med.

[52] Lutnick A (2013). Pharmacy syringe purchase test of nonprescription syringe sales in San Francisco and Los Angeles in 2010. J Urban Health: Bull N Y Acad Med.

[53] Janulis P (2012). Pharmacy nonprescription syringe distribution and HIV/AIDS: a review. J Am Pharm Assoc (2003).

[54] Deibert RJ (2006). Increased access to unrestricted pharmacy sales of syringes in Seattle–King County, Washington: structural and individual-level changes, 1996 versus 2003. Am J Public Health.

[55] Fuller CM (2007). Multilevel community-based intervention to increase access to sterile syringes among injection drug users through pharmacy sales in New York City. Am J Public Health.

[56] Katz J (2018). Why a city at the center of the opioid crisis gave up a tool to fight it. New York Times.

[57] Keyes KM (2014). Understanding the rural–urban differences in nonmedical prescription opioid use and abuse in the United States. Am J Public Health.

[58] North American Syringe Exchange Network (2018). Directory of syringe exchange programs.

