# Supplementary material for: Barriers to using new needles encountered by rural Appalachian people who inject drugs: implications for needle exchange
Source: Harm Reduct J. 2019 Apr 2;16:23. doi: 10.1186/s12954-019-0295-5 (PMC6444507; doi:10.1186/s12954-019-0295-5)
Supplement: Supplementary file 1 — Barriers to Using New Needles Questionnaire. (DOCX 24 kb) [file 12954_2019_295_MOESM1_ESM.docx]

**Barriers to Using New Needles Survey**

| Part I. For each question, please circle only one (1) response. | | | | | | |
| --- | --- | --- | --- | --- | --- | --- |
|  | **1** | **2** | **3** | **4** | **5** |  |
| 1. It takes too long to get a new needle every time I inject. | **Strongly Disagree** | **Disagree** | **Neutral** | **Agree** | **Strongly Agree** | **Not Applicable** |
| 1. It is inconvenient to get a new needle every time I inject. | **Strongly Disagree** | **Disagree** | **Neutral** | **Agree** | **Strongly Agree** | **Not Applicable** |
| 1. I often don’t want to take the time to get a new needle because my cravings or urges to use drugs are too strong. | **Strongly Disagree** | **Disagree** | **Neutral** | **Agree** | **Strongly Agree** | **Not Applicable** |
| 1. I often do not take the time to get a new needle if I am drug sick or in withdrawal. | **Strongly Disagree** | **Disagree** | **Neutral** | **Agree** | **Strongly Agree** | **Not Applicable** |
| 1. I don’t take the time to get a new needle before injecting because I can only think about getting high. | **Strongly Disagree** | **Disagree** | **Neutral** | **Agree** | **Strongly Agree** | **Not Applicable** |
| 1. I don’t take the time to get a new needle before injecting if I’m already high or drunk. | **Strongly Disagree** | **Disagree** | **Neutral** | **Agree** | **Strongly Agree** | **Not Applicable** |
| 1. The places where I inject usually do not have access to new needles. | **Strongly Disagree** | **Disagree** | **Neutral** | **Agree** | **Strongly Agree** | **Not Applicable** |
| 1. If I am in a shooting gallery (a place where people inject drugs, “dope den”, “joy popping”, etc.), I often do not use a new needle. | **Strongly Disagree** | **Disagree** | **Neutral** | **Agree** | **Strongly Agree** | **Not Applicable** |
| 1. I often do not carry new needles with me when I’m out. | **Strongly Disagree** | **Disagree** | **Neutral** | **Agree** | **Strongly Agree** | **Not Applicable** |
| 1. There isn’t a needle exchange close by for me to get needles. | **Strongly Disagree** | **Disagree** | **Neutral** | **Agree** | **Strongly Agree** | **Not Applicable** |
| 1. Pharmacies sometimes give me hassle when I try to buy needles. | **Strongly Disagree** | **Disagree** | **Neutral** | **Agree** | **Strongly Agree** | **Not Applicable** |
| 1. After I inject, I don’t prepare in advance by getting new needles ready for my next injection. | **Strongly Disagree** | **Disagree** | **Neutral** | **Agree** | **Strongly Agree** | **Not Applicable** |
| 1. It’s too expensive to buy new needles from the pharmacy for every time I inject. | **Strongly Disagree** | **Disagree** | **Neutral** | **Agree** | **Strongly Agree** | **Not Applicable** |
| 1. Feeling sad or depressed would get in the way of my using a new needle every time I inject. | **Strongly Disagree** | **Disagree** | **Neutral** | **Agree** | **Strongly Agree** | **Not Applicable** |
| 1. It is embarrassing to buy needles at the pharmacy. | **Strongly Disagree** | **Disagree** | **Neutral** | **Agree** | **Strongly Agree** | **Not Applicable** |
| 1. I worry that someone (friends, family, etc.) may see me buying needles at the pharmacy. | **Strongly Disagree** | **Disagree** | **Neutral** | **Agree** | **Strongly Agree** | **Not Applicable** |
| 1. My peers/friends would look at me funny if I used a new needle every time I inject. | **Strongly Disagree** | **Disagree** | **Neutral** | **Agree** | **Strongly Agree** | **Not Applicable** |
| 1. Having to worry about using a new needle interrupts the ritual of using. | **Strongly Disagree** | **Disagree** | **Neutral** | **Agree** | **Strongly Agree** | **Not Applicable** |
| 1. I am unlikely to use a new needle if a friend lets me borrow his or her used needle. | **Strongly Disagree** | **Disagree** | **Neutral** | **Agree** | **Strongly Agree** | **Not Applicable** |
| 1. I could get in trouble from the police if I carry needles around with me. | **Strongly Disagree** | **Disagree** | **Neutral** | **Agree** | **Strongly Agree** | **Not Applicable** |

**PART II. For the following questions, please answer or check (✓) the appropriate response.**

| 21. What is your age? (in years) _____ | 26. What is your current employment status (**check only one**)?* |
| --- | --- |
|  |  |
| 22. What is your sex? | - Employed for wages |
|  | - Self-employed |
| - - Female | - Out of work for more than 1 year |
| - - Male | - Out of work for less than 1 year |
|  | - A Homemaker |
| 23. What is the highest grade or year of school you completed?* | - A Student |
|  | - Retired |
| - Never attended school or only attended kindergarten | - Unable to work |
| - Elementary |  |
| - Some high school | 27. Are you currently homeless? |
| - High School graduate (or GED) |  |
| - Some college or technical school | - Yes |
| - College graduate | - No |
| - Some graduate or professional school |  |
| - Graduate or professional degree (for example, MS, PhD, MD) | 28. How many times do you usually inject each day?  ____ (number of injections) |
|  |  |
| 24. How would you describe your race? (**Check all that apply**)* | 29. How many times do you usually inject each week?  ____ (number of injections) |
|  |  |
| - - White | 30. How long have you been injecting? ______years |
| - - Black or African American | **OR (if less than 1 year)** ______months |
| - - Asian |  |
| - - Native Hawaiian or Pacific Islander | 31. What is your drug of choice (i.e., what drug do you most often |
| - - American Indian or Alaska Native | inject)? ___________________________________________ |
| - - Other:________________________________________ |  |
|  | 32. Are you hepatitis C positive? |
| 25. Are you Hispanic or Latino (if female, Latina)?* |  |
|  | - Yes |
| - Yes | - No |
| - No | - Don’t Know |

* Adapted from: CDC. 2014 Behavioral Risk Factor Surveillance System Questionnaire. 2013; <https://www.cdc.gov/brfss/questionnaires/pdf-ques/2014_BRFSS.pdf>. Accessed March 5th, 2015.

**THANK YOU! PLEASE RETURN THE COMPLETED SURVEY AND RECEIVE YOUR $10 SHEETZ GIFT CARD TODAY.**
